# A Rare Case of Plexiform Schwannoma of the Little Finger and Its Management: A Case Report

**DOI:** 10.7759/cureus.26391

**Published:** 2022-06-28

**Authors:** Toluwalope F Ejiyooye, Sudha Dirisanala, Hend Makky Abouzied, Syeda Sarah Mahjabeen, Taha Sajjad, Aadil Khan

**Affiliations:** 1 Family Medicine, Brooke Army Medical Center, San Antonio, USA; 2 Department of Internal Medicine, Konaseema Institute of Medical Sciences and Research Foundation, Amalapuram, IND; 3 Medicine, Thumbay University Hospital, Ajman, ARE; 4 Pathology and Laboratory Medicine, Nandamuri Taraka Rama Rao University of Health Sciences (NTRUHS), Hyderabad, IND; 5 Pathology and Laboratory Medicine, MMCH, Madinah AL Munawwara, SAU; 6 Medicine, Microsoft Virtual Machine Converter (MVMC), Phoenix, USA; 7 Department of Internal Medicine, Lala Lajpat Rai (LLR) Hospital, Kanpur, IND

**Keywords:** excision, little finger, plexiform schwannoma, fine needle aspiration cytology (fnac), myelin sheath

## Abstract

Schwannomas are tumors of the Schwann cells found in the myelin sheath. They cause 5% of all benign soft-tissue cancers, occur equally in males and females, and occur later in life. Since they remain asymptomatic, diagnosing and treating them becomes challenging; current guidelines recommend imaging followed by excision. Here, we present a case of a 19-year-old male who presents in an outpatient setting with a history of painless swelling of the fifth digit for the past four years. Past medical history and physicals are unremarkable. Microscopic findings from fine-needle aspiration cytology (FNAC) confirmed the schwannoma diagnosis, showing loosely arranged spindle cells with elongated nuclei with pointed ends dispersed within the myxoid stroma.

## Introduction

Peripheral nerve sheath tumors (PNSTs) are the most common nerve tumors of the hand and upper extremities. These tumors can be classified as benign peripheral nerve sheath tumors (BPNSTs) and malignant peripheral nerve sheath tumors (MPNSTs) [1-3]. Plexiform schwannoma is the tumor of Schwann cells of the myelin sheath and constitutes up to 5% of all benign soft tissue neoplasms, with 3% to 19% of all schwannomas [4]. The occurrence is equal in both sexes, with the prevalence most common in the third and sixth decades of life. Schwannomas remain clinically asymptomatic for years, owing to which it is challenging to investigate and manage. Clinically, it presents as localized pain and paraesthesia if the tumor begins to compress the involved nerve in the extremities. Clinical features, MRI, and ultrasonography (USG) can strengthen the diagnosis. The most updated surgical approach is tumor excision. In addition, if an accurate preoperative diagnosis is made, then a careful excision can be performed to avoid unnecessary resection of the significant nerves, which leads to better nerve recovery. Herein, we present a case of a 19-year-old male with plexiform schwannoma of the little finger in his right hand.

## Case presentation

**Figure 1:**
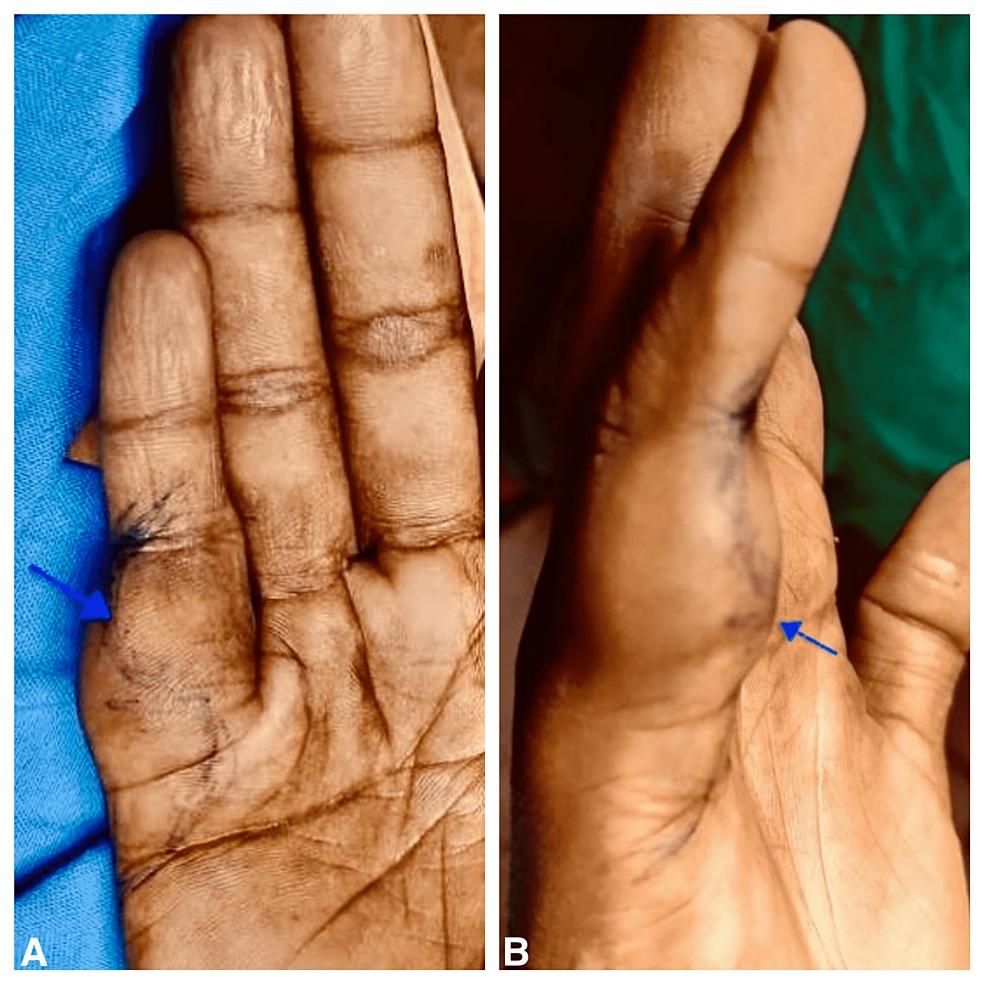
(A) and (B) Subcutaneous swelling on the ventral side of the proximal interphalangeal joint of the right little finger

We present a case of a 19-year-old male who came to the surgery outpatient department (OPD) of a public tertiary care hospital with painless swelling of his little right-hand finger for the past four years. Past medical history is insignificant for any trauma, inflammatory, or genetic disorder. On physical examination, subcutaneous swelling mainly involves the ventral side of the proximal and middle phalanges of the right hand's fifth digit, as shown in Figure 1. The swelling was immobile, nontender, and measured approximately 1.5 cm in diameter. His neurological examination reveals no loss of sensation or motor weakness, and his handgrip strength is normal. However, systemic examinations were unremarkable.

**Figure 2:**
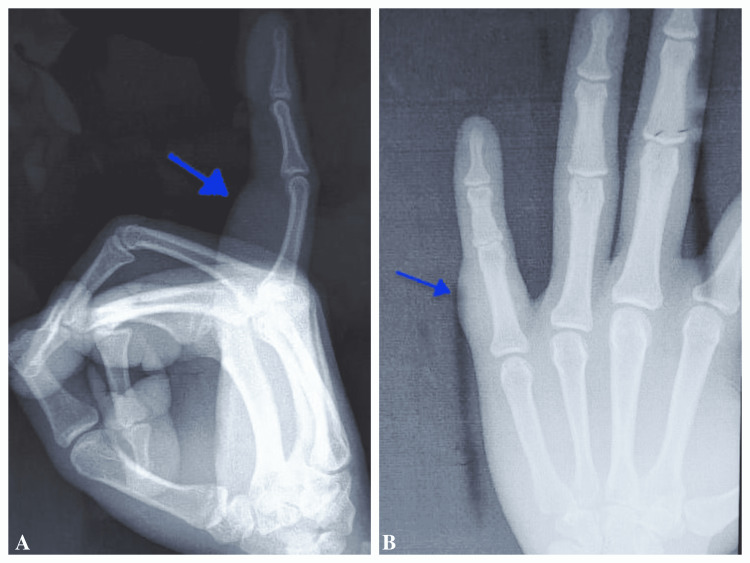
X-ray showing subcutaneous mass swelling in lateral view (A) and anteroposterior view (B)

An X-ray showed a mass located at the little right-hand finger, as shown in Figure 2. A specimen of the lesion was taken under aseptic conditions for fine-needle aspiration cytology (FNAC), and microscopic findings reveal large clustered, loosely arranged spindle cells with elongated nuclei with pointed ends. There was a wavy appearance of nuclei in places, and these cells were interspersed in a myxoid stroma. There was a palisading arrangement of nuclei forming verruca bodies in many places. These findings suggested plexiform schwannoma; however, it was later confirmed by histopathological examination.

**Figure 3:**
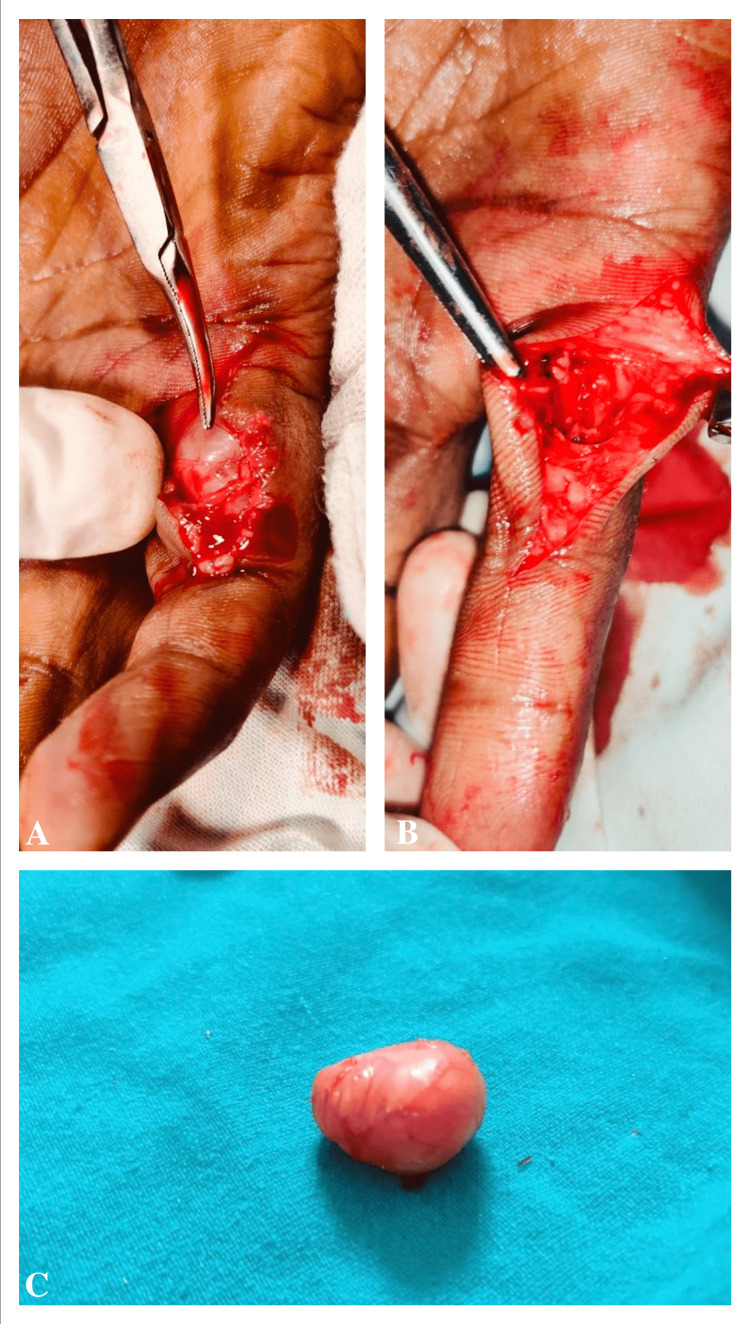
(A) Before excision, (B) after excision of the nodule, and (C) a tumor separated from the right little finger

Surgical tumor excision under local anesthesia was recommended. Informed written consent was obtained from the patient for surgery. Excision was performed without extensive dissection of tissues of the fingers, especially nerve fascicles were preserved during the operation. A nodule of 18 mm × 12 mm × 8 mm pinkish hard mass separated as shown in Figure 3.

The surgery went uneventful. Postoperative examination reveals intact movements and sensations in the right hand. In addition, the patient was prescribed antibiotics, analgesics, and multivitamins. His condition improved and he was kept under follow-up.

## Discussion

Harkin et al. discovered plexiform schwannoma in 1978. Among all schwannomas, plexiform schwannomas account for up to 5% of all schwannomas. Plexiform schwannomas are a rare benign proliferation of Schwann cells that presents as a multinodular growth pattern (plexiform) [5]. The most frequent sites of these schwannomas are in different body parts, like the head and neck region or upper extremities. Rarely presented in non-cutaneous regions like the oral cavity, vulva, deep soft tissue, breast, testis, uvea, small intestine, and colon [6]. Plexiform Schwannoma presents as a slow-growing asymptomatic solitary nodule located in the dermis and subcutaneous tissue and is rarely painful and may also present with acute rapid growth [7,8]. Symptoms may be present between 1 month and 30 years before treatment [8]. Most of these tumors are anatomically less than 2 cm in diameter [9]. Our patient presented with an immobile, non-tender swelling of a diameter of 1.5 cm in the little finger of the right hand.

The risk factors for solitary plexiform schwannoma are not entirely understood. However, the etiology of multiple tumors is multifactorial and more frequently associated with neurofibromatosis type II, schwannomatosis, Gorlin-Koutlas syndrome, and patients with a positive family history or history of trauma [10]. Plexiform schwannoma should be diagnosed anatomically and histologically because it can be mistaken for a plexiform neurofibroma. Plexiform neurofibroma is a hallmark clinical finding of neurofibromatosis and carries a potential risk of malignant transformation [11]. The histological presentation of plexiform schwannomas shows predominantly Antony A tissue consisting of compact and spindle-shaped Schwann cells in a palisading pattern or loose hypocellular area, which is the Antony B pattern and is positive for S100 expression [12]. In addition, sometimes MRI scans are often used to strengthen the diagnosis. A multinodular heterogeneous lesion characterized by tumors with low signal intensity on T1-weighted images and high intensity on T2-weighted images, and these findings suggest plexiform schwannomas. MRI scans are also helpful for preoperative planning, as they reveal the extent of the lesion and its relationship to adjacent structures [8]. Early accurate diagnosis of schwannomas results in successful treatment. In this case report, the diagnosis of plexiform schwannoma was made by cytology and later confirmed by histopathological examination.

Surgical excision is the recommended therapeutical intervention for managing cases with schwannomas in which enucleation of the tumor from the nerve sheath is done carefully without damaging the nerve and adjacent structure. In our case, we performed microsurgical dissection carefully with a surgical loupe. Ultimately, no nerve was compromised; moreover, the patient's postoperative sensory and motor function was intact in the affected digit. The patient showed improvement in response to surgery.

## Conclusions

Plexiform schwannomas of the extremities are rare tumors associated with a good prognosis due to the development of successful microsurgical dissection techniques. However, resection is unsatisfactory if the tumor is significant with the involvement of multiple sensory or motor nerve fibers. The diagnosis of plexiform schwannoma is based on imaging modalities and histopathological examination, as seen in our patient. Our patient responded well to the treatment. Further research needs to be done for better surgical therapy and pharmacotherapy for disease management and improved outcomes.
